# Assessment of Strain-Specific PrP^Sc^ Elongation Rates Revealed a Transformation of PrP^Sc^ Properties during Protein Misfolding Cyclic Amplification

**DOI:** 10.1371/journal.pone.0041210

**Published:** 2012-07-17

**Authors:** Nuria Gonzalez-Montalban, Ilia V. Baskakov

**Affiliations:** 1 Center for Biomedical Engineering and Technology, University of Maryland School of Medicine, Baltimore, Maryland, United States of America; 2 Department of Anatomy and Neurobiology, University of Maryland School of Medicine, Baltimore, Maryland, United States of America; University of Florida, United States of America

## Abstract

Prion replication is believed to consist of two components, a growth or elongation of infectious isoform of the prion protein (PrP^Sc^) particles and their fragmentation, a process that provides new replication centers. The current study introduced an experimental approach that employs Protein Misfolding Cyclic Amplification with beads (PMCAb) and relies on a series of kinetic experiments for assessing elongation rates of PrP^Sc^ particles. Four prion strains including two strains with short incubation times to disease (263K and Hyper) and two strains with very long incubation times (SSLOW and LOTSS) were tested. The elongation rate of brain-derived PrP^Sc^ was found to be strain-specific. Strains with short incubation times had higher rates than strains with long incubation times. Surprisingly, the strain-specific elongation rates increased substantially for all four strains after they were subjected to six rounds of serial PMCAb. In parallel to an increase in elongation rates, the percentages of diglycosylated PrP glycoforms increased in PMCAb-derived PrP^Sc^ comparing to those of brain-derived PrP^Sc^. These results suggest that PMCAb selects the same molecular features regardless of strain initial characteristics and that convergent evolution of PrP^Sc^ properties occurred during *in vitro* amplification. These results are consistent with the hypothesis that each prion strain is comprised of a variety of conformers or ‘quasi-species’ and that change in the prion replication environment gives selective advantage to those conformers that replicate most effectively under specific environment.

## Introduction

Prion diseases are a group of fatal age-dependent neurodegenerative maladies that can either arise spontaneously or via transmission of a prion infectious agent [1]. According to the protein-only hypothesis, the transmissible agent of prion diseases consists of a prion protein in its abnormal, β-sheet rich conformation (PrP^Sc^), which is capable of propagating itself in an autocatalytic manner by recruiting and converting the normal, cellular form of the prion protein (PrP^C^) [2]. For decades, the prion infectious agent could be replicated only using animals or cultured cells. Significant difficulties in developing an experimental system for amplification of prion infectivity *in vitro* have been inflaming debate about the biochemical nature of the prion infectious agent. In 2001, Soto and colleagues introduced the first experimental approach, referred to as Protein Misfolding Cyclic Amplification (PMCA), that permits amplification of mammalian prions *in vitro* [3]. During the past decade, PMCA fulfilled an experimental gap and became a valuable tool in prion research. The first authentic prions generated *in vitro* were produced using PMCA [4]–[7]. PMCA has been also employed for assessing the cross-species transmission barrier [8], [9], elucidating strain adaptation and interference [9], [10], exploring cofactors involved in prion replication [11]–[14] and developing ultrasensitive prion detection and titration assays [15]–[19].

Despite significant advances brought to the field with the development of PMCA, our understanding of the mechanism underlying prion replication remains limited. PMCA consists of repetitive cycles of sonication and incubation [3]. The sonication is presumably responsible for breaking PrP^Sc^ particles into smaller fragments, whereas the incubation intervals between sonication cycles are believed to be required for the growth or elongation of PrP^Sc^ particles. Co-factors including RNA and polyanions were found to stimulate prion conversion in PMCA [6], [11], [20]. On the other hand, sonication-induced degradation of RNA below a size optimal for amplification might limit the efficiency of amplification [21]. Supplementing PMCA reactions with beads (this format is referred to as PMCAb) improved the yield and the rate of amplification [22], [23]. In PMCAb, the strains with the highest conformational stability showed the largest improvements in amplification efficiency [21].

Due to technical limitations in assessing PrP^Sc^ properties in crude brain homogenate, it is difficult to gain mechanistic insight into prion replication. What are the rate limiting steps in PMCA/PMCAb? Is a PrP^Sc^ population transformed during PMCA/PMCAb? Does PMCA/ PMCAb selectively amplify PrP^Sc^ particles with certain physical features? While previous studies focused on characterization of strain-specific conformational stability and aggregation states of PrP^Sc^ [24]–[28], the current study introduces an experimental approach for assessing the dynamic properties of PrP^Sc^ and, specifically, its elongation rate. Four prion strains, two of which cause disease within very short incubation times (263K and Hyper (HY)), while the other two induce it within very long incubation times (SSLOW and LOTSS) were tested. SSLOW and LOTSS are two synthetic strains that were generated in Syrian hamsters by inoculating amyloid fibrils produced from hamster full-length recombinant prion protein [29], [30]. In a series of kinetic experiments, we showed that the PrP^Sc^ growth or elongation rate was strain-specific. Furthermore, the elongation rates of PrP^Sc^ populations were found to increase after serial PMCAb for all four strains. These results suggest that PMCAb selects the same physical features regardless of the strain initial characteristics and that convergent evolution of PrP^Sc^ properties occurred during *in vitro* amplification.

## Results

### Experimental design for assessing growth rate of PrP^Sc^ particles

**Figure 1:**
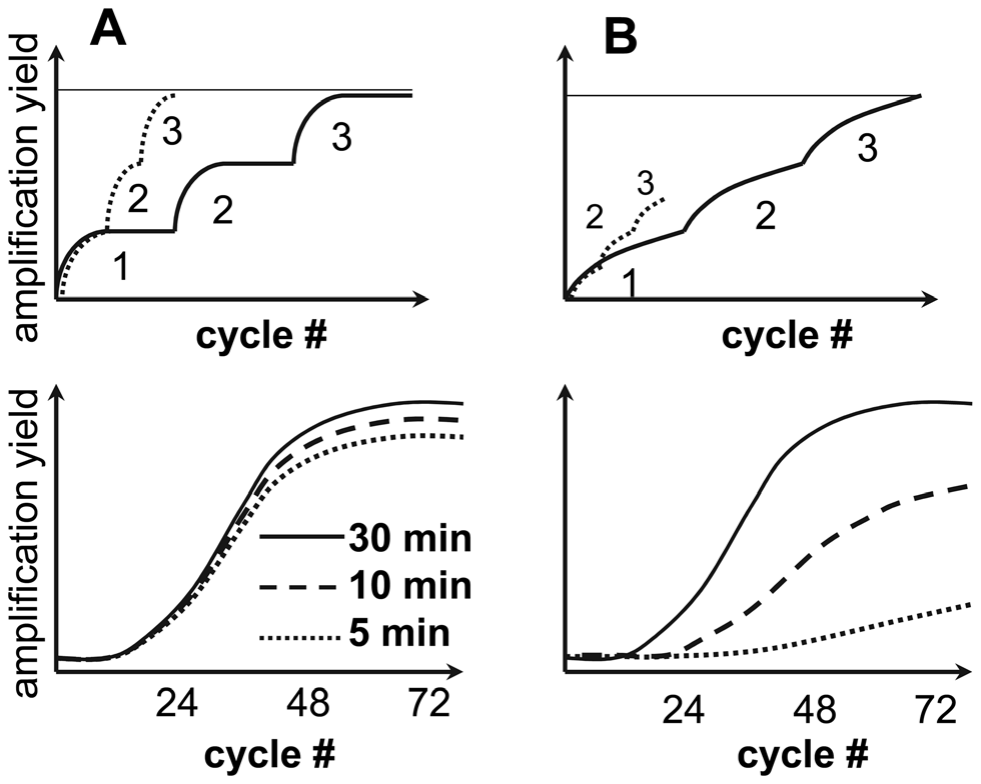
Experimental design for analysis of PrP^Sc^ elongation rate. Schematic representation of kinetics of PrP^Sc^ amplification within individual PMCAb cycles (top panels, three cycles are shown for simplicity) or within individual PMCAb rounds as a function of cycle number (bottom panels) for a strain with fast elongation rate (A) or slow elongation rate (B). The length of incubation intervals between the sonication cycles are 30 min (solid lines), 10 min (dashed lines) or 5 min (dotted lines). For a strain with a fast elongation rate, the amplification is equally effective regardless of the length of incubation intervals (A); whereas for a strain with slow elongation rate, shortening of incubation intervals leads to less effective amplification (B).

Amplification of PrP^Sc^ in PMCA or PMCAb is believed to involve two alternating steps: (i) sonication-induced fragmentation of PrP^Sc^ particles and (ii) their elongation or growth through recruiting and converting PrP^C^. In standard PMCAb format, the sonication cycles are separated by 30 minute incubation intervals, during which PrP^Sc^ particles grew in size. To test whether the PrP^Sc^ elongation rate is strain-specific, we designed a series of kinetic experiments, in which the sonication conditions were kept constant, whereas the length of incubation intervals between the sonication cycles was reduced from 30 minutes to 10 or 5 minutes (Fig. 1). If particles elongate very fast relative to the length of an incubation interval (Fig. 1A, top plot), we expect that shortening of the incubation intervals will not change the shape of a kinetic curve (Fig. 1A, bottom plot). Alternatively, if elongation is slow (Fig. 1B, to plot), it is expected that shortening of the incubation intervals will result in a less effective amplification (Fig. 1B, bottom plot). The extent to which the overall kinetic pattern changes and amplification yield drops upon shortening of the incubation intervals reports on the relative elongation rate. Due to limitations in quantitative analysis of amplification yield by Western blot, the relative estimate of an elongation rate should rely on changes in whole pattern rather than single kinetic curves or a yield at specific time points. The lag phase, if such is observed, should be considered superficial, as it is likely to reflect amplification below the detection limit of Western blot.

This model is based on several assumptions. First, it assumes that the elongation kinetics within each cycle eventually reaches a plateau, i.e. the elongation rate slows down with an increase in PrP^Sc^ particle size. While molecular reasons for plateauing are not clear, it appears that particles can not grow endlessly with the same efficiency, because sonication is required for efficient amplification. Second, we assume that strains with fast elongation rates reach plateau within the duration of an incubation interval, whereas strains with slow elongation time require longer intervals.

### Assessing the growth rate of brain-derived PrP^Sc^

**Figure 2:**
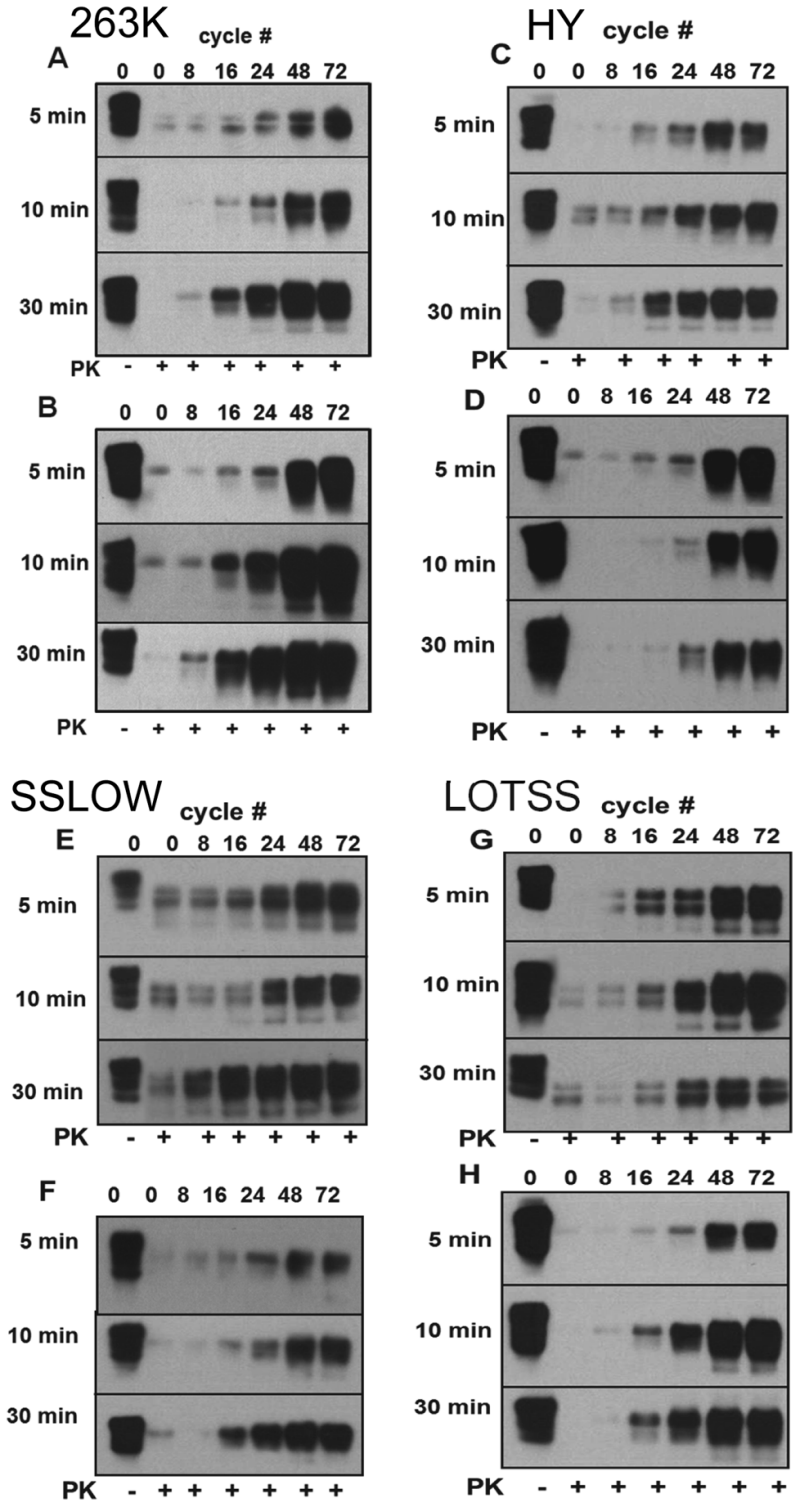
Amplification kinetics of brain-derived and PMCAb-derived PrP^Sc^. Amplification of 263K (A, B), HY (C, D), SSLOW (E, F) and LOTSS (G, H) brain-derived (A, C, E, G) or PMCAb-derived material (B, D, F, H). Brain – or PMCAb-derived materials were subjected to 8, 16, 24, 48 or 78 PMCAb cycles using three reaction formats: with 5 min, 10 min or 30 min incubation intervals between sonication cycles as indicated. For the experiments on brain-derived materials, PMCAb reactions were seeded with 10^4^ fold diluted 263K or HY brain material or 10^3^ fold diluted SSLOW or LOTSS brain material. To prepare PMCAb-derived material, six standard serial PMCAb (sPMCAb) rounds were conducted for each strain. Undigested normal brain homogenate (NBH) is provided as a reference.

For this study, we selected four strains that represent the two extremes of prion disease phenotypes, but are known to replicate in PMCAb with reasonably high efficiency. 263K and HY cause disease within very short incubation times, whereas SSLOW and LOTSS are characterized by very long incubation times of more than 300 days [29], [30]. To complete kinetic experiments within the same time frame, 10^4^ fold dilutions of 263K and HY brain materials and 10^3^ fold dilutions of SSLOW and LOTSS brain materials were used to seed PMCAb reactions. For each strain, a series of PMCAb reactions with 30, 10 or 5 min incubation intervals between sonication cycles were conducted. Aliquots were taken in the course of PMCAb reactions and amplification yield was analyzed by proteinase K (PK) digestion followed by Western blot (Fig. 2A, C, E and G). Notably, in brain-derived samples, the bands that correspond to diglycosylated PrP^Sc^ were split by a blind spot that appeared due to deposition of PK onto a membrane (Fig. S1). The splitting effect was only visible at low intensities of diglycosylated PrP^Sc^ (at 8, 16, 24 cycles) but not at high PrP^Sc^ intensities.

**Figure 3:**
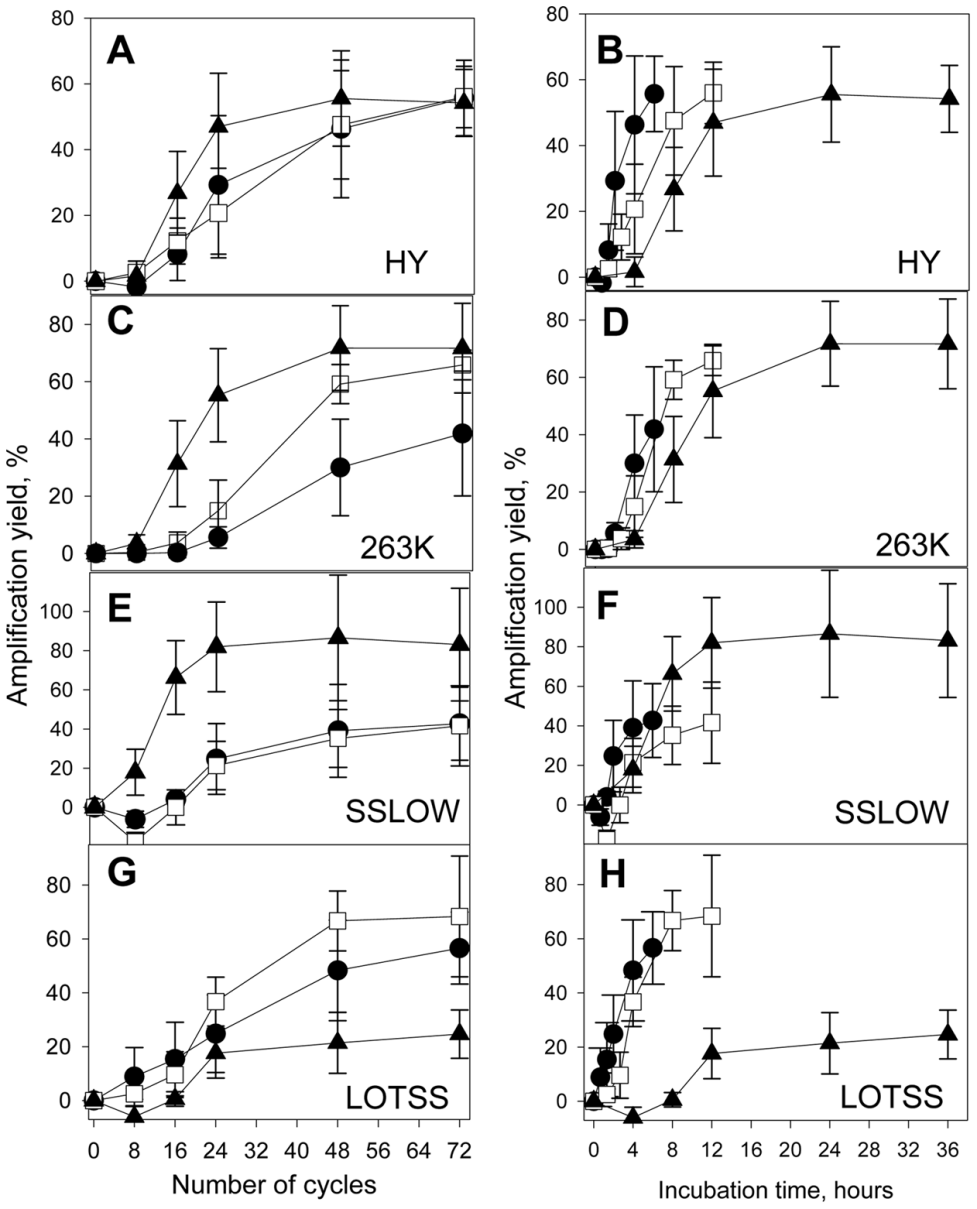
Analysis of growth rate of brain-derived PrP^Sc^. PrP^Sc^ amplification yield plotted as a function of PMCAb cycle number (left panels) or cumulative incubation interval length (right panels) obtained in PMCAb reactions consisting of incubation intervals with length of 30 min (▴), 10 min (□) or 5 min (•) between sonications and seeded with brain-derived HY (A, B), 263K (C, D), SSLOW (E, F) or LOTSS (G, H). Three independent experiments for each condition were performed and average ± SD is shown.

Two different formats were employed for presenting the same sets of data: strain-specific amplification yield was plotted as a function of cycle number (Fig. 3A, C, E and G) or as a function of cumulative incubation time intervals (Fig. 3B, D, F and H). PMCAb reactions conducted with 5 min incubation intervals were completed six times faster than the reactions conducted with 30 minute intervals, however, all PMCAb reactions consisted of 72 sonication cycles regardless of incubation interval length (Fig. 3).

**Figure 4:**
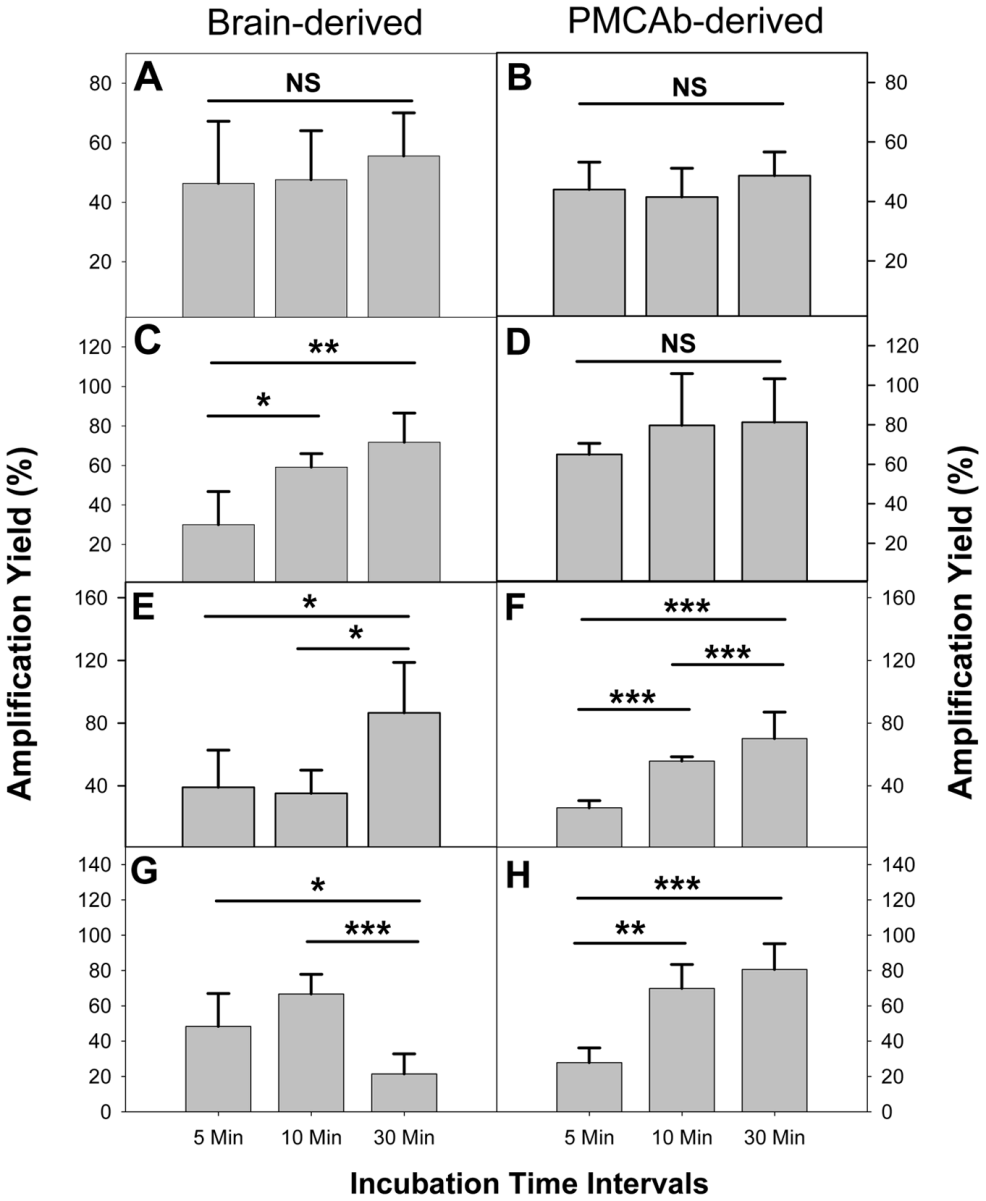
Statistical analysis of brain-derived and PMCAb-derived amplification yields. Amplification yields of brain-derived (A, C, E, D) and PMCAb-derived (B, D, F, H) PrP^Sc^ for HY (A, B), 263K (C, D), SSLOW (E, F) and LOTSS (G, H) measured at 48 cycles. Three independent experiments for each condition were performed and average ± SD is shown. Statistical analyses were performed using Student's t-test. *P<0.05; **P<0.01; ***P<0.001; NS, non-significant.

For HY, only a slight dissociation between the three kinetic curves collected at 30, 10 or 5 min incubation intervals was observed (Fig 3A). In fact, by 48 and 72 cycles, the conversion yield under all three conditions was very similar (Fig. 3A, Fig. 4A). This result indicates that incubation for only 5 min was sufficient to achieve similar outcomes as incubation for 30 minutes, which indicates a very fast elongation rate of HY PrP^Sc^.

When expressed as a function of cycle number, 263K amplification at 10 and 5 minute intervals was less efficient than that at 30 minute intervals (Fig. 3C, Fig. 4C). Furthermore, the amplification yield at 5 minute intervals was notably lower than that at 30 minute intervals (Fig. 3C). These results illustrate a more profound dissociation between the three kinetic curves for 263K PrP^Sc^ than those for HY, an indication that 263K PrP^Sc^ elongates with a slower rate than HY PrP^S^. For SSLOW, the amplification efficiency at both 10 and 5 minute intervals were significantly lower than that at 30 minute intervals (Fig. 3E, F, Fig. 4E), an indication of an even slower elongation rate for SSLOW PrP^Sc^ than that of 263K PrP^Sc^.

LOTSS showed an abnormal kinetic pattern. The amplification yield at 30 minute intervals was lower than those at 5 or 10 minute intervals (Fig. 3G, H, Fig. 4G). Such abnormal behavior could be attributed to a high conformational stability of LOTSS particles and the need for frequent sonication. Alternatively, abnormal behavior could be due to the high propensity of LOTSS PrP^Sc^ particles to rapidly cluster into large aggregates after they were fragmented by sonicaiton, a process that is likely to interfere with productive growth. Both mechanisms are consistent with frequent sonication being beneficial for LOTSS amplification.

If strain-specific elongation rate are compared based on single curves such as those at 5 and 10 min intervals but not whole kinetic profiles, LOTSS seemed to elongate faster than HY. The differences in the amount of seeds between LOTSS- and HY-seeded reactions might account for such false impression. Nevertheless, careful analysis of relative strain-specific elongation rates should rely on a comparison of whole kinetic profile, as presented in Fig. 1. To determine whether the differences in the amplification yields between 5, 10 and 30 min incubation interval curves were statistically significant, statistical analysis of a yield at the 48 cycle point was performed (Fig. 4). The analysis revealed that 263K, SSLOW and LOTSS, but not HY, showed statistically significant differences in amplification efficiency measured for 5, 10 and 30 min interval experiments (Fig. 4 A, C, E, G). The lack of difference for HY is indicative of its fastest elongation rate. In summary, the experiments that utilized brain-derived PrP^Sc^ revealed strain-specific differences in elongation kinetics. HY displayed the fastest rate, SSLOW showed the slowest rate, whereas LOTSS was characterized by abnormal pattern.

### Assessing the growth rate of PMCAb-derived PrP^Sc^

In the last few years, we persistently observed that physical properties of PrP^Sc^ population change in the course of serial PMCAb (sPMCAb) reactions. In general, PrP^Sc^ subjected to several PMCAb rounds showed more robust and efficient amplification than brain-derived PrP^Sc^. To elucidate these changes in more detail, brain-derived PrP^Sc^ from animals inoculated with HY, 263K, SSLOW or LOTSS were subjected to six standard sPMCAb rounds, then resulting PMCAb-derived PrP^Sc^ was used in a series of kinetic experiments with 30, 10 or 5 minute incubation intervals as described above (Fig. 2B, D, F, and H). Notably, the splitting effect noticed for the brain-derived diglycosylated PrP^Sc^ was not visible for PMCAb-derived samples, presumably because PMCAb-derived PrP^Sc^ normally ran slightly higher than the brain-derived PrP^Sc^.

**Figure 5:**
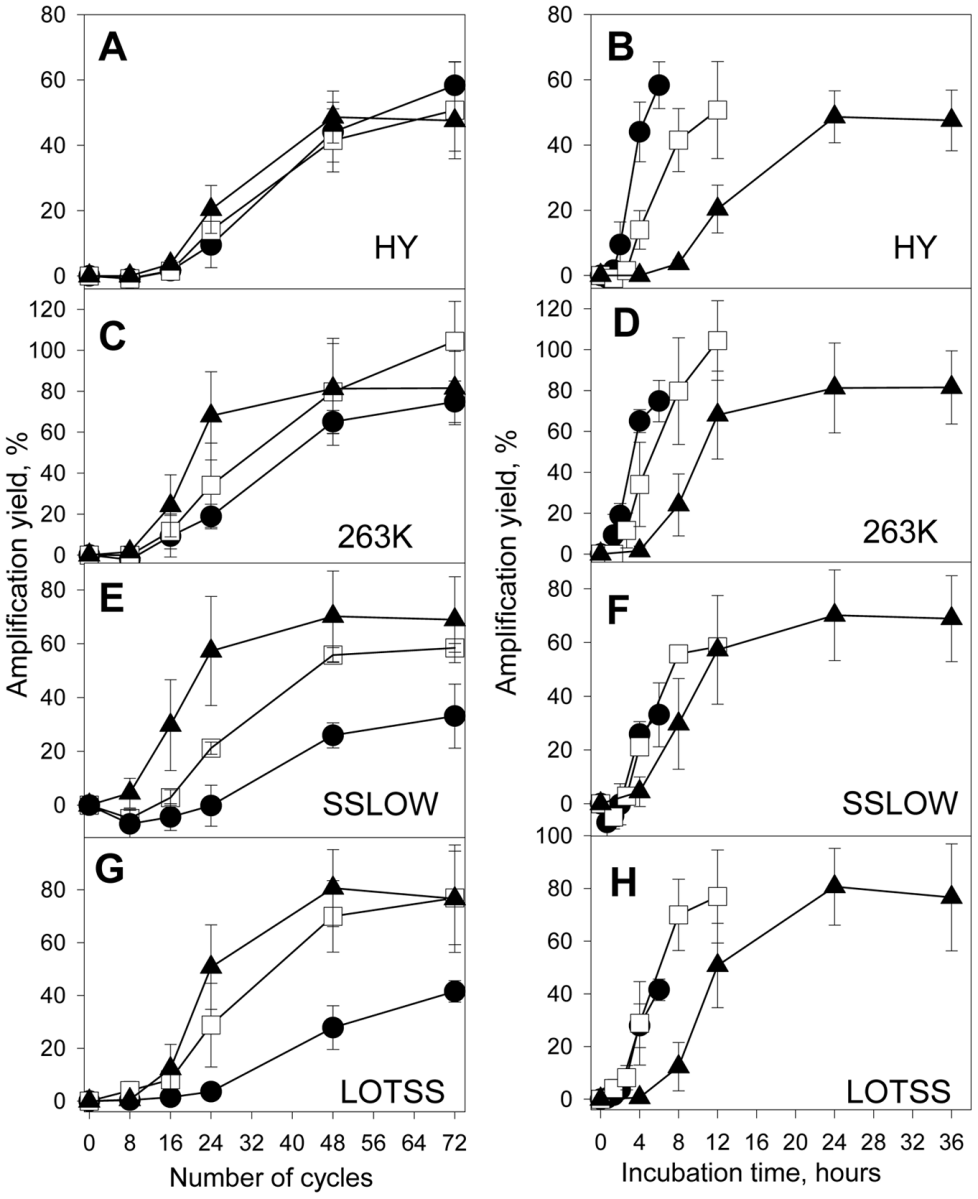
Analysis of growth rate of PMCAb-derived PrP^Sc^. PrP^Sc^ amplification yield plotted as a function of PMCAb cycle number (left panels) or cumulative incubation interval length (right panels) obtained in PMCAb reactions consisting of incubation intervals with lengths of 30 min (▴), 10 min (□) or 5 min (•) between sonications and seeded with PMCAb-derived HY (A, B), 263K (C, D), SSLOW (E, F) or LOTSS (G, H). Three independent experiments for each condition were performed and average ± SD is shown. To produce PMCAb-derived PrP^Sc^, sPMCAb were seeded with brain-derived PrP^Sc^, then six standard sPMCAb rounds were conducted.

For PMCAb-derived HY PrP^Sc^, three kinetic curves collected at 5, 10 or 30 minute intervals were fully superimposable when plotted as a function of cycle number (Fig. 5A). According to the model presented in Figure 1A, these results are indicative of a very high elongation rate for PMCAb-derived PrP^Sc^ and suggest that the rate was approximately the same as that of brain-derived PrP^Sc^. Careful analysis of the two sets of data revealed that the kinetic curves for PMCAb-derived HY PrP^Sc^ appeared to be slightly flatter than those for the reactions seeded with brain-derived HY PrP^Sc^ (compare Fig. 5A and 3A). The shape of the curves is not only determined by the PrP^Sc^ elongation rate but also seed amounts. It is difficult to normalize the seed amounts between two sets of experiments because brain-derived and PMCAb-derived PrP^Sc^ populations are likely to have different size distributions. Nevertheless, a full overlap between the three curves reports on a very fast elongation rate for HY.

For the reactions seeded with PMCAb-derived 263K PrP^Sc^, the kinetic curves collected at 5 and 10 minute intervals showed a slight dissociation from the curve at 30 minute intervals (Fig. 5C). However, in contrast to the PMCAb reactions seeded with brain-derived 263K, the final amplification yield for PMCAb-derived 263K was similar for all three conditions (compare Fig. 5C with Fig. 3C, and Fig. 4C with 4D). Again, these results indicate that the elongation rate of PMCAb-derived 263K PrP^Sc^ was higher than that of brain-derived 263K PrP^Sc^. For the reactions seeded with SSLOW or LOTSS PMCAb-derived PrP^Sc^, the amplification yield at 5 minute intervals was substantially lower than those at 10 or 30 min intervals (Fig. 5E, G), while the yield at 10 min intervals approached those at 30 min intervals after 72 cycles. These experiments demonstrate that the elongation rates of SSLOW or LOTTS PMCAb-derived PrP^Sc^ were lower than that of 263K PMCAb-derived PrP^Sc^. Nevertheless, when compared to brain-derived PrP^Sc^ (Fig. 3E), the changes in kinetic pattern of SSLOW were again consistent with an increase in the elongation rates of PMCAb-derived PrP^Sc^ relative to that of brain-derived PrP^Sc^. Moreover, the abnormal kinetic pattern found for brain-derived LOTSS PrP^Sc^ population transformed into the conventional pattern observed for the PMCAb-derived population (compare Fig. 3G with Fig. 5G). Overall, these results suggest that for each strain, PrP^Sc^ particles with fast elongation rates outcompete particles with slow elongation rates. Furthermore, for LOTSS PrP^Sc^ particles that could be easily fragmented under PMCAb conditions outcompeted and replaced particles of brain-derived PrP^Sc^ with abnormal kinetic behavior.

### PMCAb-derived PrP^Sc^ is depleted of monoglycosylated PrP

**Figure 6:**
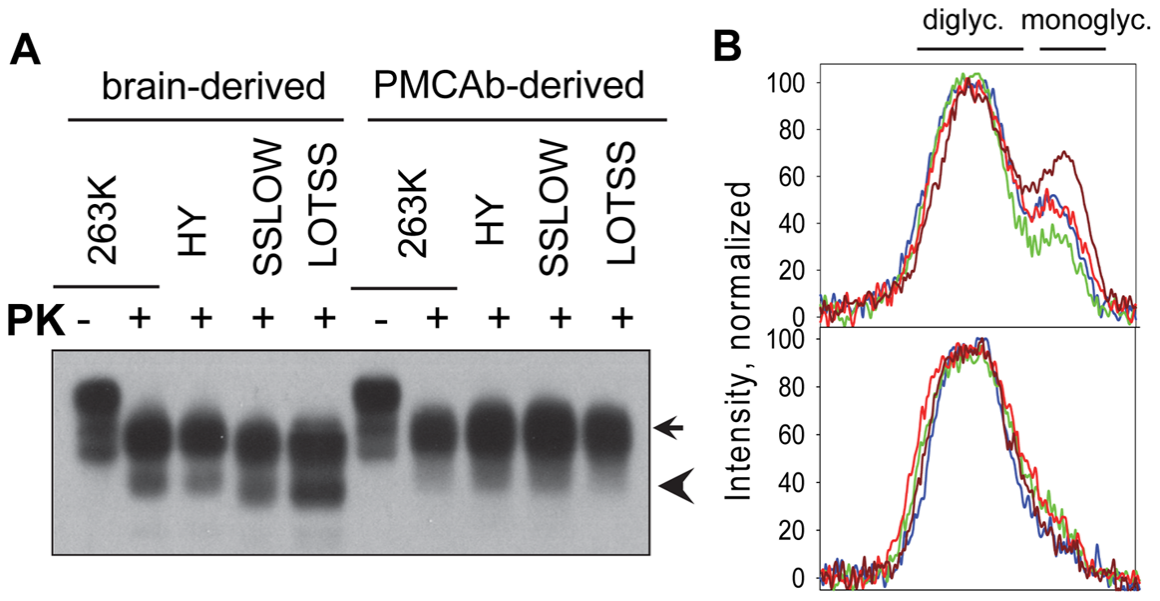
Assessing glycoform content in brain-derived and PMCAb-derived PrP^Sc^. (A) Western blot of brain-derived and PMCAb-derived PrP^Sc^ of 263K, HY, SSLOW and LOTSS. Arrow indicates diglycosylated PrP^Sc^, while arrowhead points at monoglycosylated PrP^Sc^ glycoforms. To produce PMCAb-derived PrP^Sc^, sPMCAb reactions were seeded with scrapie brain material and subjected to six standard sPMCAb rounds. Undigested NBH is provided as a reference. (B) PK resistance profiles for brain derived (top panel) and PMCAb-derived (bottom panel) PrP^Sc^ of 263K (blue), HY (green), SSLOW (red) and LOTSS (brown).

PrP^Sc^ isoforms of the hamster-adapted and synthetic hamster strains, including those used here, are known to predominantly incorporate diglycosylated and smaller amounts of monoglycosylated PrP glycoforms (Fig. 6). In multiple experiments conducted in the past, we noticed that *in vitro* amplification of hamster strains was accompanied by a relatively modest but consistent change in the PrP^Sc^ glycoform ratio. Indeed, after six PMCAb rounds, PMCAb-derived PrP^Sc^ showed a considerably lower proportion of monoglycosylated PrP glycoforms than those found in brain-derived PrP^Sc^ (Fig. 6). These results were consistent and reproducible in multiple experiments performed independently by two investigators. The difference in glycosylation status of brain- and PMCAb-derived PrP^Sc^ could not be attributed to the splitting of diglycosylated PrP^Sc^ by deposition of PK shown in Fig. S1, because monoglycosylated PrP^Sc^ band had higher mobility than both parts of diglycosylated PrP^Sc^ band. Nevertheless, the difference in glycosylation status between brain- and PMCAb-derived PrP^Sc^ could be detected only under optimal conditions for film exposure or signal intensity. Slight overexposure caused diglycosylated and monoglycosylated bands to merge, whereas under slightly underexposed conditions monoglycosylated PrP^Sc^ could not be detected as a separate band. These technical difficulties might explain the reasons why the difference in PrP^Sc^ glycosylation status was overlooked in previous studies on PMCA. A change in glycoform ratio provides an independent illustration that the composition of PrP^Sc^ populations are subject to transformation during *in vitro* amplification.

## Discussion

The current study introduced an experimental approach that relies on a series of kinetic experiments for testing the question of whether PrP^Sc^ elongation rate is strain-specific. The strains with fast elongation rates were expected to maintain the same amplification yield regardless of the length of incubation intervals between sonication cycles; whereas for the strains with slow elongation rates, the amplification yield was expected to decline with shortening of the incubation intervals (Fig. 1). The experiments with brain-derived PrP^Sc^ revealed that the elongation rate was strain-specific. Furthermore, this work showed that strain-specific elongation rates could limit the overall amplification yield in a strain-dependent manner. Among the four strains compared, HY had the highest elongation rate, SSLOW showed the lowest rate, and LOTSS exhibited abnormal kinetic behavior (Fig. 3). The abnormal kinetic pattern seen for brain-derived LOTSS PrP^Sc^ highlights the limitations of the current approach. Strains that are conformationally very stable are not susceptible to efficient fragmentation and are expected to deviate from conventional kinetic patterns. It is likely that LOTSS falls into this category. While both SSLOW and LOTSS are deposited in animal brains in form of large plaques, LOTSS was found to be conformationally more stable than SSLOW and significantly more stable than 263K [29], [30].

To test whether the physical properties of a PrP^Sc^ population change during PMCAb amplification, the strain-specific elongation rate was analyzed following six standard sPMCAb rounds. The relative ranking order in elongation rates for the four strains was preserved after they were subjected to sPMCAb. HY displayed the fastest rate, 263K had intermediate rate, while SSLOW and LOTSS showed the slowest elongation rates (Fig. 5). Remarkably, for each strain the elongation rate of PMCAb-derived populations was higher than the corresponding rate of a brain-derived PrP^Sc^ population. Moreover, the abnormal kinetic pattern observed for brain-derived LOTSS transformed into a conventional pattern after sPMCAb. Notably, after six sPMCA rounds, the kinetic pattern of 263K PrP^Sc^ became similar to that of brain-derived HY PrP^Sc^, while the patterns of SSLOW and LOTSS PrP^Sc^ resembled that of brain-derived 263K PrP^Sc^. Overall, strain-specific kinetic profiles of PMCAb-derived PrP^Sc^ were much more alike than the profiles collected for brain-derived PrP^Sc^. Taking into account previous concerns about possible cross-contamination during serial PMCA [31], we performed bioassays of PMCAb-derived PrP^Sc^. As reported previously, animals inoculated with PMCAb-derived 263K developed 263K-specific clinical symptoms and reached a terminal stage by 109±4 days postinoculation [22], whereas animals inoculated with PMCAb-derived SSLOW and LOTSS showed SSLOW- or LOTSS-specific clinical symptoms, respectively, after more than 300 days postinoculation (data not shown). The results of the current study suggest that PMCAb selects the same physical features regardless of strain initial characteristics. In other words, convergent evolution of PrP^Sc^ physical features occurred during *in vitro* amplification. Convergent evolution of the PrP^Sc^ physical properties in sPMCAb should not be mistaken for a merge of strain-specific phenotypes. In fact, previous studies demonstrated that amplification of PrP^Sc^ in sPMCA preserves the strain-specific disease phenotype [4], [6], [8], [22].

Comparison of the glycoform ratio of brain-derived and PMCAb-derived PrP^Sc^ provided an independent illustration that PrP^Sc^ populations changed during sPMCAb (Fig. 6). Again, all strains tested here showed a depletion of monoglycosylated glycoform in PMCAb-derived PrP^Sc^. This result supports the idea that the same molecular features were selected during *in vitro* amplification. We do not know whether the changes in PrP^Sc^ elongation rate was linked to a preference of PMCAb-derived PrP^Sc^ for a selective recruitment of diglycosylated PrP^C^ glyforms or whether diglycosylated hamster PrP^Sc^ has an intrinsically faster elongation rate than PrP^Sc^ composed of diglycosylated and monoglycosylated PrP glycoforms. Nevertheless, these data were consistent with previous results that stoichiometric composition of PrP^C^ glycoforms controls the conversion efficiency of prion conversion in PMCA and that the hamster strains require diglycosylated PrP^C^ for efficient amplification [32].

According to the hypothesis introduced in recent studies, each prion strain is comprised of a variety of conformers or ‘quasi-species’ [33], [34]. Change in the prion replication environment gives selective advantage to those conformers that replicate most effectively under a specific environment. The results in the current work support the view that PrP^Sc^ populations within each strain consists of ‘quasi-species’ with different elongation rates. During PMCAb, PrP^Sc^ conformers with the fastest rates accumulate more rapidly and outcompete PrP^Sc^ particles with slow rates. As a result, the physical properties of a PrP^Sc^ population change gradually during sPMCAb. This mechanism explains convergence of strain-specific kinetic profiles of PrP^Sc^ subjected to serial PMCAb. The current results also raise the possibility that relatively minor changes in PMCA/PMCAb protocols such as change in the length of incubation intervals, sonication power, or co-factor environment create different amplification conditions. Such differences are likely to give selective advantage for amplification of different PrP^Sc^ quasi-species that fit well the modified replication conditions. Therefore, the outcomes of studies that employed different PMCA/PMCAb protocols should be compared with great caution.

While PrP^Sc^ populations adapt to replicate fast under repetitive sonication cycles, PMCA- or PMCAb-amplified PrP^Sc^ might loose their fitness to replicate in cellular environments. In an animal bioassay, PMCA-amplified PrP^Sc^ was found to produce longer incubation times than brain-derived PrP^Sc^ [4], [6], [35], [36]. A decline in fitness of PMCA-adapted PrP^Sc^ to replicate and/or fragment in a cellular environment might account for longer incubation times to disease. It is likely that the mechanisms responsible for PrP^Sc^ fragmentation in PMCAb create different selective pressures in transforming PrP^Sc^ populations than the fragmentation mechanism in a cellular environment. While in PMCA or PMCAb fragmentation is caused by sonication, factors that cause fragmentation in a cell remains unknown and could involve mechanical tension, chaperones, or spontaneous fragmentation [37].

Recent studies reported contradictory results with regard to prion infectivity titers for PMCA-derived PrP^Sc^. Using HY, Shikiya and Bartz showed that the infectivity titer of PMCA-derived PrP^Sc^ was not statistically different from that of brain-derived PrP^Sc^ [36]. In contrast, the study by Klingeborn and coauthors, who employed 263K, claimed that prion infectivity titer diminishes gradually in the course of serial PMCA [35]. To explain an apparent decrease in the titer of PMCA-derived PrP^Sc^, Klingeborn et al. proposed that PMCA amplification produces infectious and non-infectious PrP^Sc^ in two competitive pathways, both of which originated from the fully infectious brain-derived PrP^Sc^ seeds [35]. Consistent with HY titration experiments, the current work revealed very minor changes in the physical properties of HY PrP^Sc^ populations during serial PMCAb. Remarkably, it appeared that brain-derived HY PrP^Sc^ was already well fit to replicate in the PMCAb environment, as shown by a very fast PMCAb elongation rate.

In contrast to HY, 263K kinetic profile was found to be a subject of a more significant transformation during sPMCAb. This result was consistent with the changes in 263K properties observed by Klingeborn and coauthors [35]. Such transformation, however, does not necessarily indicate a decline in infectivity titer as claimed by Klingeborn and coauthors [35]. Animal bioassays of PMCA-derived PrP^Sc^ were terminated at approximately 300 days postinoculation [35], a time-frame not sufficient to establish the infectivity titers by the limiting dilution approach. At PrP^Sc^ concentrations approaching limiting dilutions, a significant fraction of animals develops disease after 300 days even for strains with short incubation times. Furthermore, careful analysis of the incubation time distributions as a function of serial dilution of sPMCA-derived material suggests an alternative explanation. During sPMCA, brain-derived 263K PrP^Sc^, which is characterized by short incubation time to disease, was gradually replaced by a sPMCA-derived 263K PrP^Sc^ population that adapted well to replicate *in vitro* but had a slow replication rate in animals and was characterized by a longer incubation time to disease. Thus, the titer of sPMCA-derived 263K PrP^Sc^ form was underestimated.

In previous studies, significant effort has been spent in an attempt to establish a relationship between incubation time to disease and PrP^Sc^ physical properties such as conformational stability [21], [25], [28], [29], [38], [39]. Because conformational stability appears to control the intrinsic fragility of aggregates [37], conformational stability is likely to impact the overall rate and yield of prion amplification. The current work suggests that strain-specific differences in PrP^Sc^ elongation rate might add another dimension to a complex relationship between conformational stability, intrinsic fragility and incubation time to disease. The strains with short incubation time to disease (HY and 263K) were found to display higher elongation rates than the strains with long incubation times (SSLOW and LOTSS). However, the potential relationship between elongation rates and incubation time should be considered with a great caution, because an increase in elongation rates for PMCAb-adapted PrP^Sc^ does not lead to shorter incubation times.

The current studies illustrated that a high yield in prion amplification could be achieved by considerably shortening the length of incubation intervals in PMCAb, at least for some strains. For optimal results, the length of incubation intervals should be optimized individually for each strain. Despite of such inconvenience, the approach introduced by the current study is worthy of effort in developing rapid prion detection assays.

## Materials and Methods

### Ethics statement

This study was carried out in strict accordance with the recommendations in the Guide for the Care and Use of Laboratory Animals of the National Institutes of Health. The protocol was approved by the Institutional Animal Care and Use Committee of the University of Maryland, Baltimore (Assurance Number A32000-01; Permit Number: 0309001).

### Reagents

Hyper scrapie brain homogenate was kindly provided by Richard Bessen (Montana State University, Bozeman, MT); 263K and 10% normal brain homogenate (NBH) were kindly provided by Robert Rohwer (Veterans Affair Maryland Health Care System, Baltimore, MD). SSLOW and LOTSS scrapie brain homogenates were prepared using animals from the third passage of SSLOW and LOTSS [29], [30] with an incubation time to the terminal stage of disease of 414 and 521 days, respectively, when animals were euthanized.

### Protein misfolding cyclic amplification with beads

PMCAb procedures have been described in detail elsewhere [22]. Briefly, healthy hamsters were euthanized and immediately perfused with PBS, pH 7.4, supplemented with 5 mM EDTA. Brains were dissected, and 10% NBH (w/v) was prepared using ice-cold conversion buffer and glass/Teflon tissue grinders cooled on ice and attached to a constant torque homogenizer (Heidolph RZR2020). The composition of conversion buffer was as previously described [4]: Ca^2+^-free and Mg^2+^-free PBS, pH 7.5, supplemented with 0.15 M NaCl, 1.0% Triton and 1 tablet of Complete protease inhibitors cocktail (Roche, Cat. # 1836145) per 50 ml of conversion buffer. The resulting 10% NBH in conversion buffer was used as the substrate in PMCAb reactions. To prepare seeds, 10% scrapie brain homogenates in PBS were serially diluted in conversion buffer.

To prepare PMCAb-derived seeds, six standard sPMCAb rounds were conducted for each strain. The standard round consisted of 30 sec sonication pulses delivered at 50% or 60% efficiency applied every 30 min for 24 hours, which is 48 cycles. 10-fold dilution between serial rounds was used for all strains. To seed the kinetic experiment, the products of the 6th round of standard sPMCAb reactions were diluted 100-fold for 263K or HY and 30-fold for SSLOW or LOTSS.

For all PMCAb reactions, samples in 0.2 ml thin-wall PCR tubes (Fisher, Cat. # 14230205) supplemented with 3 Teflon beads (Small Parts, Cat. #B000FMUEXG) were placed in a floating rack inside a Misonix S-4000 microplate horn filled with 350 ml water and maintained at 37°C inside the sonicator chamber. To monitor kinetics of PrP^Sc^ replication, the sonication programs consisted of 30 sec sonication pulses delivered at 50% to 60% efficiency applied every 30 min, 10 min or 5 min in experiments with 30 min, 10 min or 5 min incubation time intervals, respectively. Aliquots were taken after 8, 16, 24, 48 or 72 cycles. To analyze PMCAb products, 10 µl of each sample were supplemented with 2.5 µμl SDS and 2.5 µl proteinase K (PK), to a final concentration of SDS and PK of 0.25% and 50 µg/ml, respectively, followed by incubation at 37°C for 1 hour. The digestion was terminated by addition of SDS-sample buffer and incubated for 10 min at 100°C. Samples were loaded onto NuPAGE 12% BisTris gels, transferred to PVDF membrane, stained with 3F4 antibody, and the Western blot was developed with Supersignal West Pico Chemiluminescent Substrate kit (Thermo Scientific, Rockford, IL).

### Data Analysis

Western blot signal intensity was assessed by densitometry and quantified using ImageJ software (NIH Image, Bethesda, MD). To calculate PrP^Sc^ amplification yield, the signal intensity of PrP^Sc^ seeds measured at PMCAb cycle #0 was subtracted from the signal intensity measured as a function of PMCAb cycle number, and the resulting signal intensity were normalized relative to undigested BH.

## Supporting Information

Figure S1 **Western blot of 263K brain material** (**0.3%**) **treated with 50 μg/ml PK.** After PK digestion, increasing concentrations of inactivated PK (from 0 to 0.75 mg/ml, marked as [PK*]) were added to samples prior to SDS-PAGE. Arrows marks a blind spot from PK that cuts dyglycosylated PrP^Sc^ band in half.
(TIF)
