# Supplementary figures and images for: Assessment of Strain-Specific PrPSc Elongation Rates Revealed a Transformation of PrPSc Properties during Protein Misfolding Cyclic Amplification
Source: PLoS One. 2012 Jul 17;7(7):e41210. doi: 10.1371/journal.pone.0041210 (PMC3398882; doi:10.1371/journal.pone.0041210)

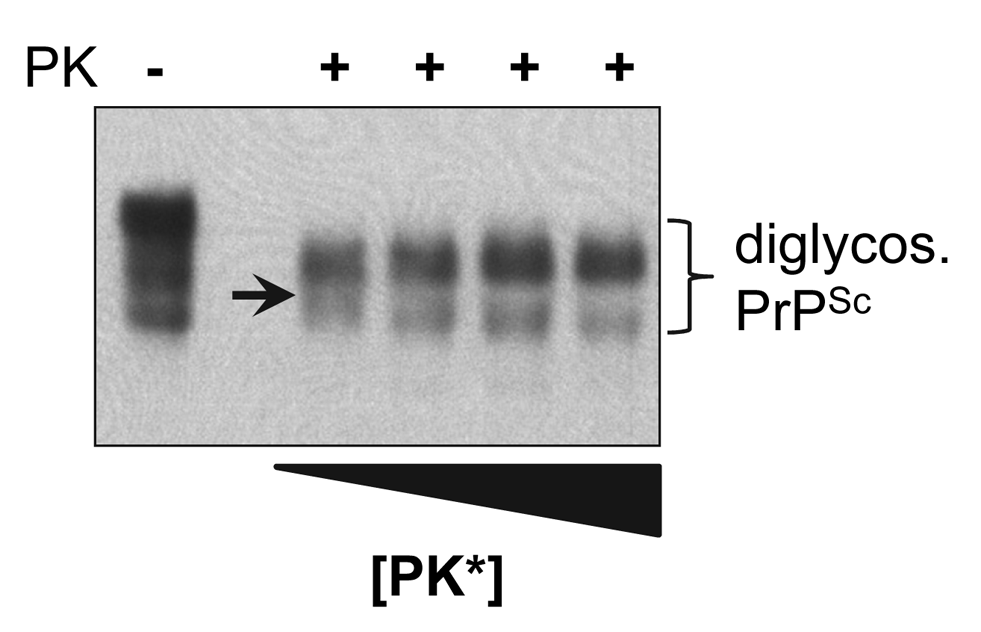

Supplement: Figure S1 — Western blot of 263K brain material (0.3%) treated with 50 μg/ml PK. After PK digestion, increasing concentrations of inactivated PK (from 0 to 0.75 mg/ml, marked as [PK*]) were added to samples prior to SDS-PAGE. Arrows marks a blind spot from PK that cuts dyglycosylated PrPSc band in half. (TIF) [file pone.0041210.s001.tif]
